# Supplementary material for: High baseline body mass index predicts recovery of CD4+ T lymphocytes for HIV/AIDS patients receiving long-term antiviral therapy
Source: PLoS One. 2022 Dec 30;17(12):e0279731. doi: 10.1371/journal.pone.0279731 (PMC9803121; doi:10.1371/journal.pone.0279731)
Supplement: S1 Checklist — (DOCX) [file pone.0279731.s001.docx]

STROBE Statement—checklist of items that should be included in reports of observational studies

|  | Item No. | Recommendation | Page  No. | Relevant text from manuscript |
| --- | --- | --- | --- | --- |
| **Title and abstract** | 1 | (*a*) Indicate the study’s design with a commonly used term in the title or the abstract | 2 | retrospective cohort study |
|  |  | (*b*) Provide in the abstract an informative and balanced summary of what was done and what was found | 2 | HIV/AIDS patients receiving ART with higher baseline BMI had better immune reconstitution and that baseline BMI could be an important predictor of immune reconstitution in patients receiving ART. Baseline BMI was not associated with virological failure, but a lower baseline BMI indicated poor viral suppression during follow-up. |
| Introduction | | | |  |
| Background/rationale | 2 | Explain the scientific background and rationale for the investigation being reported | 4 | Elucidating the relationship between BMI and CD4+ T-cell recovery is critical for a greater understanding of the biological determinants of immune reconstitution. However, conclusions about the effect of different BMI values on immune reconstitution after antiviral therapy remain inconsistent. |
| Objectives | 3 | State specific objectives, including any prespecified hypotheses | 3-4 | Body mass index (BMI) is associated with immune reconstitution in antiviral therapy patients. |
| Methods | | | |  |
| Study design | 4 | Present key elements of study design early in the paper | 4 | retrospective cohort study |
| Setting | 5 | Describe the setting, locations, and relevant dates, including periods of recruitment, exposure, follow-up, and data collection | 4-5 | HIV/AIDS patients who initiated or received antiretroviral therapy in Guangxi, China, from 2003 to 2019. The study cohort included patients who received ART between January 1, 2003, and December 31, 2019 |
| Participants | 6 | (*a*) *Cohort study*—Give the eligibility criteria, and the sources and methods of selection of participants. Describe methods of follow-up  *Case-control study*—Give the eligibility criteria, and the sources and methods of case ascertainment and control selection. Give the rationale for the choice of cases and controls  *Cross-sectional study*—Give the eligibility criteria, and the sources and methods of selection of participants | 4-5 | The inclusion criteria of patients were as follows: (a) age ≥ 18 years; (b) no pregnancy during baseline and follow-up; (c) baseline data included both weight and height indicators; (d) baseline included CD4 indicators; and (e) follow-up data had at least one CD4 record. |
|  |  | (*b*) *Cohort study*—For matched studies, give matching criteria and number of exposed and unexposed  *Case-control study*—For matched studies, give matching criteria and the number of controls per case |  |  |
| Variables | 7 | Clearly define all outcomes, exposures, predictors, potential confounders, and effect modifiers. Give diagnostic criteria, if applicable | 5 | Outcomes:CD4 count/immune reconstitution  Exposures,predictors:BMI  Potential confounders, and effect modifiers:Opportunistic infections included skin lesions, oral thrush, oral leukoplakia, persistent diarrhoea, persistent or intermittent fever, recurrent severe bacterial infections, disseminated nontuberculous infections, oesophageal candidiasis, and extrapulmonary cryptococcal infections. |
| Data sources/ measurement | 8* | For each variable of interest, give sources of data and details of methods of assessment (measurement). Describe comparability of assessment methods if there is more than one group | *5* | Data were obtained from the China AIDS Comprehensive Response Information Management System (CRIMS), including all participants' general demographic characteristics and clinical laboratory test indicators. |
| Bias | 9 | Describe any efforts to address potential sources of bias | 6 | Assuming a potential impact of viral suppression on the recovery of CD4 lymphocyte counts during follow-up, virological failure was included in the linear regression model. |
| Study size | 10 | Explain how the study size was arrived at | 5 | Fig 1 shows a flowchart of the patient inclusion criteria. |

Continued on next page

| Quantitative variables | 11 | Explain how quantitative variables were handled in the analyses. If applicable, describe which groupings were chosen and why | 5 | In our study, the grades of BMI were classified according to the standards established by the health industry standards of the People's Republic of China: underweight (BMI<18.5 kg/m2), normal (18.5 kg/m2≤BMI<24 kg/m2), overweight (24 kg/m2≤BMI<28 kg/m2), and obese (BMI≥28 kg/m2). BMI was calculated as weight (kg)/height (m)2 |
| --- | --- | --- | --- | --- |
| Statistical methods | 12 | (*a*) Describe all statistical methods, including those used to control for confounding | 6-7 | Differences in demographic and clinical characteristics of patients stratified by BMI were compared using the Pearson χ2 test (discrete variable) and the Kruskal–Wallis test (continuous variable). Univariate and multivariate linear regression analyses were performed to analyse the association between baseline variables and changes in CD4 lymphocyte counts in the first, third, and fifth years, and the normal body mass index group was used as the reference object. The discrete variables at baseline were treated as dummy variables. Assuming a potential impact of viral suppression on the recovery of CD4 lymphocyte counts during follow-up, virological failure was included in the linear regression model |
|  |  | (*b*) Describe any methods used to examine subgroups and interactions |  |  |
|  |  | (*c*) Explain how missing data were addressed |  |  |
|  |  | (*d*) *Cohort study*—If applicable, explain how loss to follow-up was addressed  *Case-control study*—If applicable, explain how matching of cases and controls was addressed  *Cross-sectional study*—If applicable, describe analytical methods taking account of sampling strategy |  |  |
|  |  | (*e*) Describe any sensitivity analyses | 7 | To avoid bias caused by differences between BMI levels, sensitivity analysis was used to further verify the reliability of the results, and trend tests were performed by BMI categories. |
| Results | | | | |
| Participants | 13* | (a) Report numbers of individuals at each stage of study—eg numbers potentially eligible, examined for eligibility, confirmed eligible, included in the study, completing follow-up, and analysed | 7-8 | Statistical analysis was performed on 6139 patients (28167.4 person-years) |
|  |  | (b) Give reasons for non-participation at each stage |  |  |
|  |  | (c) Consider use of a flow diagram | 5 | Fig 1 shows a flowchart of the patient inclusion criteria. |
| Descriptive data | 14* | (a) Give characteristics of study participants (eg demographic, clinical, social) and information on exposures and potential confounders | 7-8 | A total of 1451 (23.6%) patients were underweight, 3882 (63.2%) were normal weight, 682 (11.1%) were overweight, and 124 (2%) were obese. The majority of patients receiving ART were 4183 males (68.1%) |
|  |  | (b) Indicate number of participants with missing data for each variable of interest |  |  |
|  |  | (c) *Cohort study*—Summarise follow-up time (eg, average and total amount) | 7 | Statistical analysis was performed on 6139 patients (28167.4 person-years) |
| Outcome data | 15* | *Cohort study*—Report numbers of outcome events or summary measures over time | *8-9* | changes in CD4 lymphocyte counts during follow-up;  CD4 lymphocyte count <200 cells/μL recovery rate after starting antiviral therapy and its influencing factors |
|  |  | *Case-control study—*Report numbers in each exposure category, or summary measures of exposure |  |  |
|  |  | *Cross-sectional study—*Report numbers of outcome events or summary measures |  |  |
| Main results | 16 | (*a*) Give unadjusted estimates and, if applicable, confounder-adjusted estimates and their precision (eg, 95% confidence interval). Make clear which confounders were adjusted for and why they were included | 8-9 | The results of Table 2 showed that after adjusting for BMI, which was related to the change in CD4 lymphocyte count, with normal weight as the reference group, the gain in CD4+ T cells in overweight patients in the first year was not significant (8.70(-6.17-23.56), P=0.250), and the patients with higher BMI in the subsequent period had higher changes in CD4 lymphocyte count (P<0.05), all of which were statistically significant. Obese patients had significant CD4+ cell gain (1st year: 72.27 (38.96-105.58), P<0.001; 3rd year: 78.25 (28.40-128.09), P = 0.002; 5th year: 113.37 (48.37-178.37), P<0.001). |
|  |  | (*b*) Report category boundaries when continuous variables were categorized | 8-9 | the patients with higher BMI in the subsequent period had higher changes in CD4 lymphocyte count (P<0.05), all of which were statistically significant. Obese patients had significant CD4+ cell gain (1st year: 72.27 (38.96-105.58), P<0.001; 3rd year: 78.25 (28.40-128.09), P = 0.002; 5th year: 113.37 (48.37-178.37), P<0.001). |
|  |  | (*c*) If relevant, consider translating estimates of relative risk into absolute risk for a meaningful time period |  |  |

Continued on next page

| Other analyses | 17 | Report other analyses done—eg analyses of subgroups and interactions, and sensitivity analyses | 11 | For the purpose of sensitivity analysis, we also handled BMI as a categorical variable, and changes in the corresponding effect sizes (1, 1.03, 1.45, 1.68) of BMI categories indicated that with increasing BMI, the risk trend of CD4 lymphocyte count returning to ≥ 500 cells/μL gradually increased (P for trend < 0.001). |
| --- | --- | --- | --- | --- |
| Discussion | | | | |
| Key results | 18 | Summarise key results with reference to study objectives | 12 | We found that the higher the baseline BMI was, the better the effect of immune reconstitution was for patients undergoing ART. The gain in CD4+ T cells increased with baseline BMI, and with a longer follow-up, the gain in CD4+ T cells in obese patients changed significantly |
| Limitations | 19 | Discuss limitations of the study, taking into account sources of potential bias or imprecision. Discuss both direction and magnitude of any potential bias | 14 | Our study benefits from a large sample, but there are still some limitations. First, because it was a retrospective cohort study, there may have been selection bias, resulting in significant differences in baseline characteristics. Second, there were fewer obese people in the study, approximately 2%. However, a sensitivity analysis showed the positive effect of BMI and immune reconstitution in obese patients. Third, this study only illustrates the association between BMI level and immune reconstitution but cannot prove a causal relationship between the two. Finally, these data lack follow-up BMI values, and thus, no further analysis of the relationship between updated BMI and immune reconstitution is provided. |
| Interpretation | 20 | Give a cautious overall interpretation of results considering objectives, limitations, multiplicity of analyses, results from similar studies, and other relevant evidence | 14 | However, for HIV-infected patients, obesity may have a protective effect on immune reconstitution. It may be that higher leptin levels promote immune recovery. |
| Generalisability | 21 | Discuss the generalisability (external validity) of the study results |  |  |
| Other information | |  | | |
| Funding | 22 | Give the source of funding and the role of the funders for the present study and, if applicable, for the original study on which the present article is based | 15-16 | This study received support from the National Natural Science Foundation of China (Grant Nos. 81803295 and 81760602), the Natural Science Foundation of Guangxi (2018GXNSFAA138031), the “Thirteenth Five-Year” National Major Science and Technology Projects (2018ZX10715008–002 and 2018ZX10302104–001), the Innovation Project of Guangxi Graduate Education (YCSW2021143), and the Opening topic fund of Guangxi Key Laboratory of AIDS Prevention and Treatment (No.gklapt 201902). |

*Give information separately for cases and controls in case-control studies and, if applicable, for exposed and unexposed groups in cohort and cross-sectional studies.

**Note:** An Explanation and Elaboration article discusses each checklist item and gives methodological background and published examples of transparent reporting. The STROBE checklist is best used in conjunction with this article (freely available on the Web sites of PLoS Medicine at http://www.plosmedicine.org/, Annals of Internal Medicine at http://www.annals.org/, and Epidemiology at http://www.epidem.com/). Information on the STROBE Initiative is available at www.strobe-statement.org.
