# Supplementary material for: High baseline body mass index predicts recovery of CD4+ T lymphocytes for HIV/AIDS patients receiving long-term antiviral therapy
Source: PLoS One. 2022 Dec 30;17(12):e0279731. doi: 10.1371/journal.pone.0279731 (PMC9803121; doi:10.1371/journal.pone.0279731)
Supplement: S1 Fig — (PDF) [file pone.0279731.s002.pdf]

S1 Fig. Association of Baseline BMI with Poor Viral Suppression

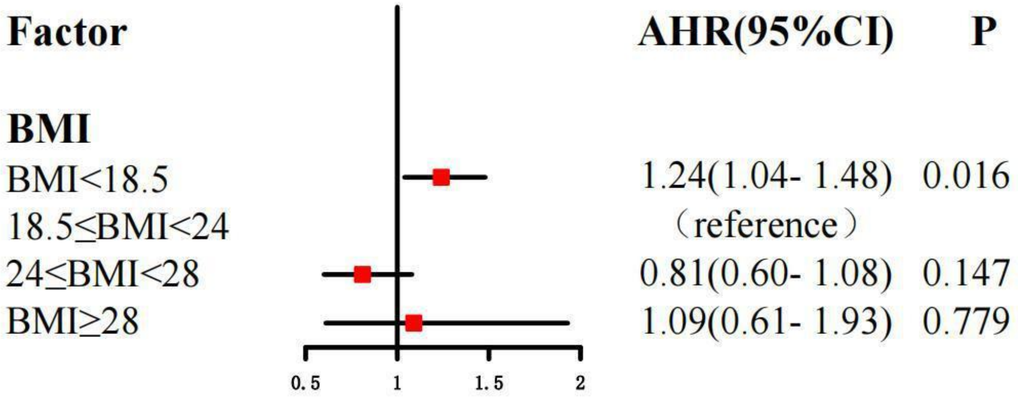

The Cox regression model was adjusted for significant covariates from the univariate model, with BMI as the categorical variable. BMI Normal group as reference group.
