# Supplementary material for: High baseline body mass index predicts recovery of CD4+ T lymphocytes for HIV/AIDS patients receiving long-term antiviral therapy
Source: PLoS One. 2022 Dec 30;17(12):e0279731. doi: 10.1371/journal.pone.0279731 (PMC9803121; doi:10.1371/journal.pone.0279731)
Supplement: S1 Table — (DOCX) [file pone.0279731.s003.docx]

**S1 Table.** **Univariate and Multivariate Analysis of CD4 Change of HIV Positive and AIDS with or without Symptoms**

|  | **Univariable analysis** | | **Multivariable analysis** | |
| --- | --- | --- | --- | --- |
| **Whether** **the patients who have developed AIDS ? (ref: HIV positive patients)** | **95%CI** | **P** | **95%CI** | **P** |
| The 1st year | -10.42(-20.76, -0.08) | 0.048 | -10.82(-24.12, 2.48) | 0.111 |
| The 3rd year | 17.71 (3.98, 31.44) | 0.011 | -0.29 (-17.59, 17.01) | 0.974 |
| The 5th year | 24.09 (6.08, 42.1) | 0.009 | -11.68 (-33.93, 10.58) | 0.304 |
| The 8th year | 65.81 (34.49, 97.12) | <0.001 | -2.49 (-39.59, 34.6) | 0.895 |
| **In AIDS patients, whether the patients have AIDS defining symptoms? (ref: No)** |  |  |  |  |
| The 1st year | 3.56 (-16.16, 23.29) | 0.723 | 3.04 (-17.2, 23.27) | 0.768 |
| The 3rd year | -4.33 (-37.93, 29.27) | 0.800 | -9 (-41.04, 23.04) | 0.582 |
| The 5th year | 53.64 (8.66, 98.63) | 0.019 | 43.95 (-0.73, 88.63) | 0.054 |
| The 8th year | 106.67 (-11.5, 224.85) | 0.077 | 146.7 (-28.29, 265.11) | 0.113 |
