# Supplementary material for: High baseline body mass index predicts recovery of CD4+ T lymphocytes for HIV/AIDS patients receiving long-term antiviral therapy
Source: PLoS One. 2022 Dec 30;17(12):e0279731. doi: 10.1371/journal.pone.0279731 (PMC9803121; doi:10.1371/journal.pone.0279731)
Supplement: S2 Table — (DOCX) [file pone.0279731.s004.docx]

**S2 Table. Association between BMI and CD4 lymphocyte count recovery (≥500cells/μL) in different models**

| **Variable** | **Crude Model** | **Model I** | **Model II** |
| --- | --- | --- | --- |
|  | **HR (95%CI))** | **HR (95%CI)** | **HR (95%CI)** |
| BMI | 1.04(1.02, 1.06) | 1.05(1.03, 1.07) | 1.03(1.01, 1.05) |
| **BMI categorie** |  |  |  |
| BMI<18.5 | Ref | Ref | Ref |
| 18.5≤BMI<24 | 1.09(0.96, 1.24) | 1.16(1.02, 1.33) | 1.03(0.96, 1.18) |
|  |  |  |  |
| 24≤BMI<28 | 1.53(1.23, 1.91) | 1.70(1.37, 2.12) | 1.45(1.16, 1.83) |
|  |  |  |  |
| BMI≥28 | 2.15(1.38, 3.33) | 2.20(1.41, 3.41) | 1.68(1.07, 2.63) |
|  |  |  |  |
| **P for trend** | <0.001 | <0.001 | <0.001 |
