# Supplementary material for: High baseline body mass index predicts recovery of CD4+ T lymphocytes for HIV/AIDS patients receiving long-term antiviral therapy
Source: PLoS One. 2022 Dec 30;17(12):e0279731. doi: 10.1371/journal.pone.0279731 (PMC9803121; doi:10.1371/journal.pone.0279731)
Supplement: S3 Table — (DOCX) [file pone.0279731.s005.docx]

**S3 Table. Factors associated with virologic failure**

| Variable | Univariate | | Multivariate | |
| --- | --- | --- | --- | --- |
|  | HR(95%CI) | P | AHR(95%CI) | P |
| **BMI** |  |  |  |  |
| BMI<18.5 | 1.22(0.94, 1.58) | 0.146 |  |  |
| 18.5≤BMI<24（reference） | 1 | 1 |  |  |
| 24≤BMI<28 | 0.72(0.47, 1.10) | 0.127 |  |  |
| BMI≥28 | 0.35(0.08, 1.42) | 0.142 |  |  |
| **Gender（ref:Male）** | 0.73(0.57, 0.95) | 0.018 | 0.90(0.68, 1.17) | 0.418 |
| **Age** | 0.99(0.98, 1.01) | 0.367 |  |  |
| **Marital status** |  |  |  |  |
| unmarried（reference） | 1 | 1 | 1 | 1 |
| married or living together | 0.64(0.49, 0.84) | 0.001 | **0.69(0.53, 0.90)** | **0.007** |
| divorced or separated | 0.79(0.50, 1.26) | 0.326 | 0.76(0.47, 1.21) | 0.247 |
| Widowed and others | 1.15(0.75, 1.77) | 0.509 | 1.21(0.78, 1.88) | 0.396 |
| **Transmission route** |  |  |  |  |
| blood transmission（reference） | 1 | 1 | 1 |  |
| Intravenous drug use | 0.99(0.31, 3.25) | 0.997 | 0.91(0.28, 2.99) | 0.876 |
| sexually transmitted | 0.32(0.10, 1.01) | 0.052 | 0.33(0.10, 1.02) | 0.055 |
| other | 0.15(0.03, 0.74) | 0.02 | **0.18(0.04, 0.88)** | **0.035** |
| **WHO clinical stage** |  |  |  |  |
| Ⅰ（reference） | 1 | 1 | 1 |  |
| Ⅱ | 1.52(1.03, 2.25) | 0.035 | 1.12(0.74, 1.72) | 0.592 |
| Ⅲ | 1.41(0.99, 2.02) | 0.058 | 0.84(0.53, 1.34) | 0.465 |
| Ⅳ | 1.97(1.51, 2.57) | <0.001 | 0.98(0.63, 1.52) | 0.935 |
| **CD4+ T-cell count** | 0.99(0.99, 1.00) | <0.001 | 1.00(0.99, 1.00) | 0.230 |
| **VL(log10 copies/ml)** | 1.12(0.99, 1.26) | 0.069 | 1.00(0.89, 1.22) | 0.980 |
| **Cotrimoxazole use before baseline（ref:yes）** | 0.49(0.39, 0.62) | <0.001 | **0.64(0.47, 0.86)** | **0.003** |
| **Mtb infection in the recent year** |  |  |  |  |
| Yes（reference） | 1 | 1 |  |  |
| No | 0.81(0.59, 1.12) | 0.206 |  |  |
| Unknown | 1.54(0.48, 4.96) | 0.469 |  |  |
| **Initial treatment plan** |  |  |  |  |
| PI-based（reference） | 1 | 1 |  |  |
| NNRTI-based | 1.06(0.70, 1.61) | 0.785 |  |  |
| NRTI-only | 1.02(0.24, 4.33) | 0.974 |  |  |
| Other | - | - |  |  |
| **Opportunistic infection（ref:No）** | 1.8(1.43, 2.27) | <0.001 | 1.31(0.91, 1.87) | 0.142 |
| **Time from diagnosis to treatment delay （ref:≤3 months）** | 0.96(0.74, 1.25) | 0.77 |  |  |
