# Supplementary material for: High baseline body mass index predicts recovery of CD4+ T lymphocytes for HIV/AIDS patients receiving long-term antiviral therapy
Source: PLoS One. 2022 Dec 30;17(12):e0279731. doi: 10.1371/journal.pone.0279731 (PMC9803121; doi:10.1371/journal.pone.0279731)
Supplement: S4 Table — (DOCX) [file pone.0279731.s006.docx]

**S4 Table . Factors associated with Poor Viral Suppression**

| Variable | Univariate | | Multivariate | |
| --- | --- | --- | --- | --- |
|  | HR(95%CI) | P | AHR(95%CI) | P |
| **BMI** |  |  |  |  |
| BMI<18.5 | 1.41(1.19, 1.67) | <0.001 | 1.24(1.04, 1.48) | 0.016 |
| 18.5≤BMI<24（reference） | 1 |  | 1.00 |  |
| 24≤BMI<28 | 0.70(0.52, 0.93) | 0.014 | 0.81(0.60, 1.08) | 0.147 |
| BMI≥28 | 0.91(0.51, 1.62) | 0.748 | 1.09(0.61, 1.93) | 0.779 |
| **Gender（**ref:Male**）** | 0.91(0.77, 1.06) | 0.228 | - | - |
| **Age** | 1.00(1.00, 1.01) | 0.947 | - | - |
| **Marital status** |  |  |  |  |
| unmarried（reference） | 1 |  | 1.00 |  |
| married or living together | 0.77(0.65, 0.92) | 0.004 | **0.81(0.68, 0.97)** | **0.023** |
| divorced or separated | 0.96(0.72, 1.30) | 0.81 | 0.92(0.68, 1.24) | 0.570 |
| Widowed and others | 0.99(0.73, 1.34) | 0.937 | 0.96(0.71, 1.31) | 0.807 |
| **Transmission route** |  |  |  |  |
| blood transmission（reference） | 1 |  | 1.00 |  |
| Intravenous drug use | 1.44(0.53, 3.97) | 0.477 | 1.28(0.46, 3.53) | 0.636 |
| sexually transmitted | 0.63(0.24, 1.69) | 0.361 | 0.62(0.23, 1.67) | 0.348 |
| other | 0.29(0.09, 0.97) | 0.045 | 0.32(0.10, 1.05) | 0.061 |
| **WHO clinical stage** |  |  |  |  |
| Ⅰ（reference） | 1 |  | 1.00 |  |
| Ⅱ | 1.58(1.24, 2.03) | <0.001 | 1.32(1.02, 1.72) | 0.036 |
| Ⅲ | 1.41(1.12, 1.76) | 0.003 | 0.97(0.73, 1.30) | 0.847 |
| Ⅳ | 1.70(1.42, 2.03) | <0.001 | 1.05(0.79, 1.39) | 0.735 |
| **CD4+ T-cell count** | 0.99(0.99, 1.00) | <0.001 | 0.99(0.99, 1.00) | 0.002 |
| **VL(log10 copies/ml)** | 1.06(0.99, 1.15) | 0.1 | - | - |
| **Cotrimoxazole use before baseline（**ref:yes**）** | 0.59(0.51, 0.69) | <0.001 | **0.79(0.65, 0.96)** | **0.015** |
| **Mtb infection in the recent year** |  |  |  |  |
| Yes（reference） | 1 |  | 1.00 |  |
| No | 0.78(0.63, 0.95) | 0.014 | 1.05(0.83, 1.31) | 0.701 |
| Unknown | 0.99(0.40, 2.43) | 0.983 | 1.22(0.49, 3.01) | 0.667 |
| **Initial treatment plan** |  |  |  |  |
| PI-based（reference） | 1 |  |  |  |
| NNRTI-based | 1.14(0.87, 1.51) | 0.345 | - | - |
| NRTI-only | 1.13(0.45, 2.81) | 0.801 | - | - |
| Other |  |  | - | - |
| **Opportunistic infection（**ref:No**）** | 1.63(1.40, 1.89) | <0.001 | 1.22(0.97, 1.53) | 0.092 |
| **Time from diagnosis to treatment delay （**ref:≤3 months**）** | 0.94(0.80, 1.12) | 0.505 | - | - |
