# Supplementary material for: High baseline body mass index predicts recovery of CD4+ T lymphocytes for HIV/AIDS patients receiving long-term antiviral therapy
Source: PLoS One. 2022 Dec 30;17(12):e0279731. doi: 10.1371/journal.pone.0279731 (PMC9803121; doi:10.1371/journal.pone.0279731)
Supplement: Attachment — Submitted filename: Response to Reviewers.docx [file pone.0279731.s007.docx]

Article Title: High baseline body mass index predicts recovery of CD4+ T lymphocytes for HIV/AIDS patients receiving long-term antiviral therapy

Dear editors and reviewers,

Thank you for your further feedback. Please see below for our responses to your comments and concerns, listed in their original order of appearance; your comments are shown in bold, while our responses are shown in regular font type.

As per before, if you have any further questions, please do not hesitate to let us know.

Sincerely,

Chuanyi Ning

**Editor’s comments:**

**When submitting your revision, we need you to address these additional requirements.**

**1. Please ensure that your manuscript meets PLOS ONE's style requirements, including those for file naming. The PLOS ONE style templates can be found at**

**https://journals.plos.org/plosone/s/file?id=wjVg/PLOSOne_formatting_sample_main_body.pdf and** 

**<https://journals.plos.org/plosone/s/file?id=ba62/PLOSOne_formatting_sample_title_authors_affiliations.pdf>**

**Response:**

Thank you for the comment. We have double checked and modified the format of the manuscript as required.

**2. Thank you for stating in your Funding Statement:**

**"This study received support from the National Natural Science Foundation of China (Grant Nos. 81803295 and 81760602), the Natural Science Foundation of Guangxi (2018GXNSFAA138031), the “Thirteenth Five-Year” National Major Science and Technology Projects (2018ZX10715008–002 and 2018ZX10302104–001), the Innovation Project of Guangxi Graduate Education (YCSW2021143), and the Opening topic fund of Guangxi Key Laboratory of AIDS Prevention and Treatment (No.gklapt 201902)."**

**Please provide an amended statement that declares *all* the funding or sources of support (whether external or internal to your organization) received during this study, as detailed online in our guide for authors at** **http://journals.plos.org/plosone/s/submit-now.  Please also include the statement “****There was no additional external funding received for this study.” in your updated Funding Statement.**

**Please include your amended Funding** **Statement within your cover letter. We will change the online submission form on your behalf.**

Response:

Thank you for the critique. We have included the funding statement in the cover letter, as well as the updated manuscript.

3. **Thank you for stating the following financial disclosure:**

**"This study received support from the National Natural Science Foundation of China (Grant Nos. 81803295 and 81760602), the Natural Science Foundation of Guangxi (2018GXNSFAA138031), the “Thirteenth Five-Year” National Major Science and Technology Projects (2018ZX10715008–002 and 2018ZX10302104–001), the Innovation Project of Guangxi Graduate Education (YCSW2021143), and the Opening topic fund of Guangxi Key Laboratory of AIDS Prevention and Treatment (No.gklapt 201902)."**

**Please state what role the funders took in the study. If the funders had no role, please state: ""****The funders had no role in study design, data collection and analysis, decision to publish, or preparation of the manuscript.""**

**If this statement is not correct you must amend it as needed.**

**Please include this amended** **Role of Funder statement in your cover letter; we will change the online submission form on your behalf.**

Response:

Thank you for the critique. We have included the Role of Funder statement in the cover letter, as well as the updated manuscript.

4. **Your ethics statement should only appear in the Methods section of your manuscript. If your ethics statement is written in any section besides the Methods, please move it to the Methods section and delete it from any other section. Please ensure that your ethics statement is included in your manuscript, as the ethics statement entered into the online submission form will not be published alongside your manuscript.**

Response:

Thank you for the critique. We have moved the ethical statement to the Methods section.

5. **Please review your reference list to ensure that it is complete and correct. If you have cited papers that have been** **retracted, please include the rationale for doing so in the manuscript text, or remove these references and replace them with relevant current references. Any changes to the reference list should be mentioned in the** **rebuttal letter that accompanies your revised manuscript. If you need to cite a retracted article, indicate the article’s retracted status in the References list and also include a citation and full reference for the retraction notice.**

Response:

Thank you for the critique. We have replaced the incorrect reference in the revised version. Please let us know if you still have any question.

The details are as follows:

***Original citation:*** [7]STD and AIDS Prevention and Control Center CCfDCaP. National Free HIV Antiretroviral Drug Treatment Handbook (4th Edition): People's Medical Publishing House; 2016.

***Replaced Citation:*** [7][Chinese guidelines for diagnosis and treatment of HIV/AIDS (2018)]. Zhonghua nei ke za zhi. 2018;57(12):867-84.

**Reviewers’ comments:**

**Reviewer #1: Have the authors made all data underlying the findings in their manuscript fully available?**

Response:

Thank you for the critique. Many thanks to the editor for your efforts to this manuscript. We have the right to access the raw data and no administrative permissions is required. But the raw data is not publicly available because of ethical and legal reasons.

**The results state that Most patient were followed for ≤8 years. Furthermore, the authors also present that CD4+ T cells continued to increase with the length of follow-up. The authors however did not present results from the follow up after 8 years in the univariate and multivariate regression models even though this was used for the Kaplan Meier curves. it would be important to review which variables remained statistically significant across 1, 3, 5 and also 8 years of follow up.**

Response:

Thank you for the critique. Most patients were followed up for ≤8 years, 114 patients (about 1.9%) were followed up for more than 8 years, and only 79 patients were able to calculate the change of CD4 lymphocyte count. In order to avoid the low efficiency of statistical test, our analysis data were not included in the patients who had been followed up for more than 8 years. Therefore, we supplemented the 8-year follow-up data to the updated table 2 according your suggestions. The data showed that age, the occurrence of virologic failure, and sex were related to the change in CD4+ T cells during the 1st, 3rd, 5th and 8th years of follow-up. Taking the fifth year as an example, older age [-3.36 (-4.06, -2.65), P<0.001] and the occurrence of virologic failure [-115.81 (-150.32, -81.29), P<0.001] were negative factors for CD4+ T-cell gain. CD4+ T-cell concentration was significantly increased in females compared with males [48.28 (30.15, 66.41), P<0.001].

**Reviewer #2: REVIEW FOR MANUSCRIPT TITLED “HIGH BASELINE BODY MASS INDEX PREDICTS RECOVERY OF CD4+ T LYMPHOCYTES FOR HIV/AIDS PATIENTS RECEIVING LONG-TERM ANTIVIRAL THERAPY”**

**In this manuscript, the authors performed a database review of HIV-positive individuals to ascertain the relationship between baseline BMI and CD4 T cell count recovery in patients who are receiving ARVs for a period of 17 years.**

**The long duration of the review provided a robust database to obtain data. This is a favorable aspect of the study. The statistical input was quite detailed. The findings from the work tend to suggest a direct relationship between CD4 T cell count and BMI ie patients with higher BMI tend to have better recovery of CD4+ lymphocytes Findings from this work will influence key aspects of clinical decision-making in the care and management of patients living with HIV**

**However, the authors should provide clarity on the following aspects:**

**1. The author should distinguish between** **patients who** **have developed AIDs based on Clinical and laboratory criteria and those** **who are HIV positive.**

Response:

Thank you very much for pointing this out. We have added the analysis to compare the CD4 changes between the HIV positive, AIDS with AIDS defining symptoms and AIDS without AIDS defining symptoms according your comment #1 and comment #2. The data analysis results showed that there was no correlation between each group for CD4 changes, and the difference was not statistically significant. We added these results in the supplementary materials, please see the Table S1. Please let us know if you still have question.

**Table S1.** **Univariate and Multivariate Analysis of CD4 Change of HIV Positive and AIDS with or without Symptoms**

|  | **Univariable analysis** | | **Multivariable analysis** | |
| --- | --- | --- | --- | --- |
| **Whether** **the patients who have developed AIDS ? (ref: HIV positive patients)** | **95%CI** | **P** | **95%CI** | **P** |
| The 1st year | -10.42(-20.76, -0.08) | 0.048 | -10.82(-24.12, 2.48) | 0.111 |
| The 3rd year | 17.71 (3.98, 31.44) | 0.011 | -0.29 (-17.59, 17.01) | 0.974 |
| The 5th year | 24.09 (6.08, 42.1) | 0.009 | -11.68 (-33.93, 10.58) | 0.304 |
| The 8th year | 65.81 (34.49, 97.12) | <0.001 | -2.49 (-39.59, 34.6) | 0.895 |
| **In AIDS patients, whether the patients have AIDS defining symptoms? (ref: No)** |  |  |  |  |
| The 1st year | 3.56 (-16.16, 23.29) | 0.723 | 3.04 (-17.2, 23.27) | 0.768 |
| The 3rd year | -4.33 (-37.93, 29.27) | 0.800 | -9 (-41.04, 23.04) | 0.582 |
| The 5th year | 53.64 (8.66, 98.63) | 0.019 | 43.95 (-0.73, 88.63) | 0.054 |
| The 8th year | 106.67 (-11.5, 224.85) | 0.077 | 146.7 (-28.29, 265.11) | 0.113 |

2. **There should be a statistical sub-analysis based upon this classification. This will help to put findings from the study in a proper perspective. For instance, it should be clear from the analysis if the recovery of CD4 T cells and T lymphocytes is better in** **patients WITHOUT** **AIDs defining symptoms** **versus** **p****atients** **WITH AIDs defining symptoms.**

Response:

Thank you very much for your comments. As mentioned above, please see the Table S1 above. We selected patients defined as AIDS, and divided them into two groups according to whether they have AIDS defining symptoms or not. We used a linear regression model to analyze and compare the changes of CD4 in patients without AIDS defining symptoms versus patients with AIDS defining symptoms. Results also shown that there was no statistical difference between these two groups. Please see more details in the Table S1.
