# Supplementary material for: High baseline body mass index predicts recovery of CD4+ T lymphocytes for HIV/AIDS patients receiving long-term antiviral therapy
Source: PLoS One. 2022 Dec 30;17(12):e0279731. doi: 10.1371/journal.pone.0279731 (PMC9803121; doi:10.1371/journal.pone.0279731)
Supplement: Attachment — Submitted filename: Response to Reviewers - new.docx [file pone.0279731.s008.docx]

Article Title: High baseline body mass index predicts recovery of CD4+ T lymphocytes for HIV/AIDS patients receiving long-term antiviral therapy

Dear editors and reviewers,

Thank you for your further feedback. Please see below for our responses to your comments and concerns, listed in their original order of appearance; your comments are shown in bold, while our responses are shown in regular font type.

As per before, if you have any further questions, please do not hesitate to let us know.

Sincerely,

Chuanyi Ning

**Editor’s comments:**

**Journal Requirements:**

**Please review your reference list to ensure that it is complete and correct. If you have cited papers that have been** **retracted, please include the rationale for doing so in the manuscript text, or remove these references and replace them with relevant current references. Any changes to the reference list should be mentioned in the** **rebuttal letter that accompanies your revised manuscript. If you need to cite a retracted article, indicate the article’s retracted status in the References list and also include a citation and full reference for the retraction notice.**

Response:

Thank you for the critique. We have updated the reference style in the revised edition and replaced the incorrect reference. Please let us know if you still have any question.

The details are as follows:

***Original citation:*** [34]Sørensen TI, Echwald S, Holm JC. Leptin in obesity. BMJ (Clinical research ed). 1996;313(7063):953-4.

***Replaced Citation:*** [34]Zimmet P, Hodge A, Nicolson M, Staten M, de Courten M, Moore J, et al. Serum leptin concentration, obesity, and insulin resistance in Western Samoans: cross sectional study. BMJ (Clinical research ed). 1996;313(7063):965-9. Epub 1996/10/19. doi: 10.1136/bmj.313.7063.965. PubMed PMID: 8892415; PubMed Central PMCID: PMCPMC2352310.

We have fully dealt with the comments of the two reviewers in the last round of review and have been recognized by the two reviewers. Here we have updated the references and hope to get your reply.
